# Construction and protective evaluation of a recombinant attenuated *Salmonella* vaccine delivering *Mycoplasma synoviae* antigens

**DOI:** 10.1080/21505594.2025.2545554

**Published:** 2025-08-08

**Authors:** Guihua Zhang, Lejiabao Han, Yuying Zhao, Zewei Li, Yu-An Li, Quan Li, Shifeng Wang, Huoying Shi

**Affiliations:** a College of Veterinary Medicine, Yangzhou University, Yangzhou, China; b Jiangsu Co‐Innovation Center for the Prevention and Control of Important Animal Infectious Diseases and Zoonoses, Yangzhou, China; c Department of Infectious Diseases and Immunology, College of Veterinary Medicine, University of Florida, Gainesville, FL, USA; d Joint International Research Laboratory of Agriculture and Agri‐Product Safety, Yangzhou University (JIRLAAPS), Yangzhou, China

**Keywords:** *Mycoplasma synoviae*, recombinant attenuated *Salmonella* vaccine, immune response, protection efficiency, prevention

## Abstract

*Mycoplasma synoviae* (*M. synoviae*) is a highly detrimental avian pathogen that has resulted in substantial economic losses within the global poultry industry. The existing vaccines, presently in use, fail to offer comprehensive protection. The objective of this study was to create recombinant attenuated *Salmonella* vaccine (RASV) strains, namely rSC0130(pS-rRS01790), rSC0130(pS-rBMP), rSC0130(pS-rGrpE), rSC0130(pS-rRS00900), and rSC0130(pS-rRS00275), expressing RS01790, BMP, GrpE, RS00900, and RS00275 proteins, and verify their protective effects. Assessed candidate vaccines for their growth characteristics, plasmid stability, and capability to synthesize exogenous antigens. The results demonstrated that the expression of exogenous antigens had no detrimental impact on the growth performance of the candidate vaccine strain. Additionally, the plasmid remained stable and continued to express through the 50th generation, confirming its long-term stability within the strain. Furthermore, all five antigens were successfully synthesized within the candidate vaccines. The immune protection results demonstrated that candidate vaccine immunization induced specific humoral, mucosal, and cellular immune responses. The growth performance of the rSC0130(pS-rRS01790), rSC0130(pS-rBMP), and rSC0130(pS-rGrpE) immunized groups exhibited significant recovery, and the air sac lesion scores were significantly lower than those of the challenge control group and the rSC0130(pS0018) control group. All candidate vaccines effectively reduced the colonization of *M. synoviae* in the pharynx and mitigated damage to the tracheal mucosa. These results underscore the potential of the RASV carrying the *M. synoviae* antigen as a promising approach for the development of vaccines against *M. synoviae* and suggest that RASV could serve as a novel strategy for prevention and control of *M. synoviae* infections.

## Introduction

*M. synoviae* is a significant avian pathogen known to induce both acute and chronic diseases in poultry, encompassing conditions such as synovitis, air sac inflammation, and eggshell abnormalities, thereby resulting in growth retardation and reduced egg production [1]. *M. synoviae* infection is a year-round occurrence and leads to substantial economic losses. The extent of this infection has extended to encompass regions across Europe, Asia, Africa, Oceania, and the Americas, ultimately surpassing the impact of *Mycoplasma gallisepticum* in the poultry industry [2–5].

The MS-H attenuated vaccine had been registered in multiple countries [6,7], serving to minimize both the vertical and horizontal transmission of *M. synoviae* in poultry and leading to a reduction in the occurrence of air sac lesions and abnormalities in eggshell formation caused by *M. synoviae* infection in poultry [8,9]. Nonetheless, the utilization of the MS-H vaccine poses challenges for the comprehensive monitoring of *M. synoviae* [10], does not effectively prevent the colonization of wild strains within the host [11–13], and reports indicate that live vaccines may mutate under field conditions [14,15]. Furthermore, the MS-H vaccine necessitates administration to individuals lacking *M. synoviae* infection, significantly constraining its broad applicability [9].

Inactivated vaccines, while characterized by stability and ease of transport, have the drawback of eliciting a comparatively delayed immune response. The growth rate of *M. synoviae* is notably sluggish, necessitating a prolonged cultivation period. The supplementation of the culture medium with essential nutrients like NAD and animal serum contributes to the heightened cost associated with vaccine production [16,17]. Consequently, there is a requirement for the use of innovative approaches for the development of a novel vaccine targeting *M. synoviae*.

Cellular and mucosal immune responses play a crucial role in the host’s defense against *M. synoviae* [18,19]. The ability of vaccines to elicit strong cellular and mucosal immune responses depends on the incorporation of high-quality adjuvants. The recombinant attenuated *Salmonella* vaccine (RASV) distinguishes itself as an outstanding antigen delivery platform that can concurrently function as an effective adjuvant [20]. It offers a cost-effective and needle-free approach for delivering exogenous antigens, significantly enhancing the immunogenicity and cost-efficiency of vaccines [21]. Thus far, it has been demonstrated that RASV effectively delivers antigens derived from bacteria, viruses, and parasites, thereby eliciting protective immune responses [22–24]. *Salmonella*, as a vector, has the ability to express various Toll-like receptor agonists, including flagellin, lipopolysaccharide (LPS), and lipoproteins. This expression significantly enhances both cellular and mucosal immune responses to exogenous antigens [25–27]. Live bacterial carriers must allocate their limited metabolic resources to synthesize an adequate amount of exogenous antigens, ensuring the elicitation of the necessary immune response without compromising colonization. However, excessive colonization can give rise to concerns regarding biological safety [28]. Attenuated live Salmonella vaccines have the potential to enhance vaccine safety. Nevertheless, an excessive level of attenuation may compromise their immunogenicity [29]. To address these concerns, our recent research developed the *Salmonella* mRNA interference regulation vector (SIRV) rSC0130 [30]. This vector contains the MazF enzyme, an interfering enzyme from Escherichia coli that cleaves single-stranded mRNA with a 5”-ACA-3” sequence. Therefore, we designed the gene encoding the target protein to lack the ACA nucleotide sequence (ACA-). When the MazF enzyme function is activated, mRNA from other genes containing the ACA sequence (ACA+) is degraded, leading to a significant increase in the relative expression of the target protein.

RS01790 (a putative sugar ABC transporter lipoprotein), BMP (a substrate-binding protein of the BMP family ABC transporter), GrpE (a nucleotide exchange factor), RS00900 (a putative nuclease), and RS00275 (an uncharacterized protein) are proteins that were previously screened in the laboratory and demonstrated good immunogenicity as subunit vaccines against *M. synoviae* [31]. This study employed RASV rSC0130 as a carrier to create rSC0130(pS-rRS01790), rSC0130(pS-rBMP), rSC0130(pS-rGrpE), rSC0130(pS-rRS00900), and rSC0130(pS-rRS00275) candidate vaccines for delivering *M. synoviae* antigens. The study conducted assessments of the growth characteristics, stability, and the capacity to synthesize exogenous antigens in the candidate vaccine strains. Additionally, the study evaluated their ability to induce immune responses and provide protection against *M. synoviae* infection using a chicken model, offering novel insights for the development of *M. synoviae* vaccines.

## Materials and methods

### Animals and ethics statement

Specific pathogen-free (SPF) chicken embryos were purchased from Jiangsu Boehringer Ingerhaviton Biotechnology Co., Ltd. (Nantong, China). The chickens used in this study were hatched from SPF chicken embryos in our laboratory. The chickens were housed in standard animal facilities, provided with unlimited access to water and food. All experiments conducted at Yangzhou University were approved by the Jiangsu Administrative Committee for Laboratory Animals (permission number SYXK (SU) 2021‐0027). The present study was approved (approval no. 202302010) by Ethics Committee of Yangzhou University (Yangzhou, China). The procedures strictly followed the guidelines of Yangzhou University Animal Welfare and Ethics, adhering to international regulations.

### Plasmids, bacterial strains, and growth conditions

The strains and plasmids utilized are shown in Table 1. *M. synoviae* strain rSC0200 was grown in the Frey Medium Modified Base (Solarbio, Beijing, China) supplemented with horse sera (10%), NAD (10 µg/mL), cysteine hydrochloride hydrate (10 µg/mL), arginine (40 µg/mL), and 800,000 IU of penicillin G (Shenggong, Shanghai, China). *E. coli* strain χ7213 was maintained in Luria-Bertani (LB) liquid medium or on LB agar at 37°C supplemented with 2,6-diaminopimelic acid (DAP; 50 µg/mL). While *E. coli* strain χ7213 carried plasmids pS-RS01790, pS-BMP, pS-GrpE, pS-RS00900, and pS-RS00275 (expression vectors) or pS0018 (empty vector) were cultured in LB liquid medium or on LB agar at 37°C. Recombinant attenuated *Salmonella* Typhimurium (*S*. Typhimurium) rSC0130, which was constructed by our laboratory (unpublished, UK-1Δ*relA::araC* P_araBAD_ *lacI* TT, Δ*pmi*, Δ*endA*::TT *araC* P_araBAD_ *mazE* TT, Δ*cysG*:P_lac_ *mazF*, Δ*asdA*) was maintained in LB liquid medium or on LB agar at 37°C supplemented with DAP, arabinose (200 µg/mL), and mannose (200 µg/mL). rSC0130 carried plasmids pS-RS01790, pS-BMP, pS-GrpE, pS-RS00900, and pS-RS00275 or pS0018 were cultured in LB broth supplemented with arabinose and mannose.

**Table 1.** Bacterial strains and plasmids used in this study.

Strains and plasmid	Characteristics	Sources and references
*E. coli* strains		
DH5α	For Construction the recombinant plasmids	Invitrogen
χ7213	thi1 thr1 leuB6 fhuA21 lacY1 glnV44 asdA4 recA1 RP4 2Tc:Mu pir	Provided by Dr.Roy Curtiss III
*S*. Typhimurium		
rSC0130	ΔrelA:araC PBAD lacI TT ΔendA:araC PBAD mazE TTΔmanA ΔcysG:Plac mazF ΔasdA	Stored in our lab
*Mycoplasma synoviae*		
rSC0200	Wild-type	[31]
Plasmids		
pS0018	Plasmid Asd+; pBR ori, β-lactamase signal sequence-based periplasmic secretion plasmid	[26]
pMD19-T	Cloning vector; Ampr	TaKaRa
pS-rRS01790	pS0018 with rRS01790	This study
pS-rBMP	pS0018 with rBMP	This study
pS-rGrpE	pS0018 with rGrpE	This study
pS-rRS00900	pS0018 with rRS00900	This study
pS-rRS00275	pS0018 with rRS00275	This study

Ampr, Ampicillin resistance.

### Construction of vaccine strains and detection of proteins expression

Table 2 primers used in this study. The genes RS01790, BMP, GrpE, RS00900, and RS00275 from the *M. synoviae* were amplified from plasmids previously constructed [31] and integrated into the *EcoR* I and *Sal* I (*Takara*, Dalian, China) restriction enzyme sites of the plasmid pS0018 backbone, turned into plasmids pS-RS01790, pS-BMP, pS-GrpE, pS-RS00900, and pS-RS00275. The vector control plasmid pS0018, along with the pS-RS01790, pS-BMP, pS-GrpE, pS-RS00900, and pS-RS00275 plasmids encoding *M. synoviae* antigen biosynthesis genes, were transferred to asd-deficient *S*. Typhimurium rSC0130 via electroporation. The resulting transformants were selected on LB agar plates containing arabinose and mannose lacking DAP. The expression of RS01790, BMP, GrpE, RS00900, and RS00275 was verified by western blot using anti-RS01790, -BMP, -GrpE, -RS00900, and -RS00275 antisera (1:1000), respectively, as previously described [32].

**Table 2.** The primers information.

Primer name	Sequences (5’–3’)	Target gene
pS-rRS01790-F	CCGGAATTCAAGCTGAGCAAAAAATTCCTGC	rRS01790
pS-rRS01790-R	ACGCGTCGACTTAGCTGCGATCAACAAACTGATC	
pS-rBMP-F	CCGGAATTCAAGAAGAAGTTCATCCTGCCGA	rBMP
pS-rBMP-R	ACGCGTCGACTTACTGCTGGGTGGTATTGGTCT	
pS-rGrpE-F	CCGGAATTCAAGGAAAATATTCTGAAGCGCC	rGrpE
pS-rGrpE-R	ACGCGTCGACTTATTTTTTATTTTTAACCACAACCACTT	
pS-rRS00900-F	CCGGAATTCAAGAAGATCAAGAAGATCGCACTGG	rRS00900
pS-rRS00900-R	ACGCGTCGACTTACTTGATGGTTTCATGCTTAATTTT	
pS-rRS00275-F	CCGGAATTCAAGAAGTTCGAGTTCCTGCTGC	rRS00275
pS-rRS00275-R	ACGCGTCGACTTACTTCTTGGTGGCTGCCAG	

Underlined nucleotides denote enzyme restriction sites

### Determination of plasmid stability and bacterial growth curves

Continuous culture was performed for 50 generations by diluting the bacteria at a ratio of 1:100 and transferring the diluted suspension to LB broth supplemented with arabinose and mannose. This approach aimed to assess the stability of plasmids pS-RS01790, pS-BMP, pS-GrpE, pS-RS00900, and pS-RS00275 in strain rSC0130. Cultures were allowed to grow for 12 h which was counted as one generation. At the 50th generation, plasmids pS-RS01790, pS-BMP, pS-GrpE, pS-RS00900, and pS-RS00275 were extracted for analysis. Their verification was carried out using double enzymatic digestion, while the presence of rSC0130 was confirmed by PCR with the appropriate primers.

The growth rates of rSC0130(pS-RS01790), rSC0130(pS-BMP), rSC0130(pS-GrpE), rSC0130(pS-RS00900), and rSC0130(pS-RS00275) strains were assessed in LB broth supplemented with arabinose and mannose. The strains were diluted 1:100 in 50 mL of fresh LB liquid and incubated at 37°C. The OD_600_ was measured at 2-h intervals for 12 h using a spectrophotometer (Eppendorf AG) to generate the growth curves.

### Salmonella subcellular fractionation

In this study, the supernatant, periplasmic, and cytoplasmic fractions were extracted from five candidate vaccine strains to assess the subcellular localization of the synthesized proteins RS01790, BMP, GrpE, RS00900, and RS00275, following a previously described method with modifications [33]. The bacterial cultures were grown to an OD_600_ of 0.8 and induced with 0.5 mM IPTG for 3 h. To ensure consistency, bacterial density for each strain was normalized based on absorbance at OD_600_. The 50 mL bacterial culture was subjected to centrifugation at 7,000 × *g* for 10 min. The supernatant fluid was then filtered through a 0.45 μm-pore-size filter, and proteins were subsequently precipitated using 10% trichloroacetic acid for 60 min at 4°C. The resulting precipitated proteins were harvested by centrifugation at 16,000 × *g* for 60 min at 4°C, followed by two washes with acetone. Finally, the proteins were resuspended in 4 mL of 20 mM Tris-HCl (pH 8.6). The cell pellets were initially resuspended in 800 μl of 100 mM Tris-HCl buffer, which included 500 mM sucrose and 0.5 mM EDTA. Following this, 40 μL of lysozyme (4 mg/mL) was added, and without delay, 3.2 mL of 50 mM Tris-HCl buffer, containing 250 mM sucrose, 0.25 mM EDTA, and 2.5 mM MgCl_2_, was added. The mixture was gently agitated and then incubated for 15 min in an ice bath. Subsequently, the cells were separated by centrifugation at 7,000 × *g* for 6 min, and the supernatant was filtered through a 0.45 μm-pore-size filter to obtain the periplasmic fraction. Meanwhile, the remaining cells were resuspended in 4 mL of 20 mM Tris-HCl, disrupted using a cell disrupter, and the resulting lysate was collected. The resulting proteins were separated by SDS-PAGE using a 12% polyacrylamide gel, and a subsequent western blot analysis was performed [34].

### Immunization and challenge in chickens

One-day-old SPF chicks were randomly divided into eight groups (*n* = 10): challenge control, blank control, rSC0130(pS0018), rSC0130(pS-RS01790), rSC0130(pS-BMP), rSC0130(pS-GrpE), rSC0130(pS-RS00900), and rSC0130(pS-RS00275) for assessing the immune protection efficiency. For immunization [32,35], the strains were grown overnight in LB broth supplemented with arabinose and mannose at 37°C. Afterward, the overnight cultures of the strains were diluted 1:100 and incubated at 37°C until they reached an OD_600_ of 0.8 to 0.9. Subsequently, the cells were collected through centrifugation and washed twice with PBS. A bacterial inoculum of 2 ± 0.3 × 10 [9] CFU was administered to each chick (half orally and half through nostrils), followed by a booster at day 14. All chicks were immunized twice, while the blank control group was administered with PBS. Blood samples were collected 2 weeks after each inoculation, and the serum samples were stored at −80°C after centrifugation. Two weeks after the booster inoculation, all chickens except the blank control group were challenged with a broth culture of rSC0200 (9 × 10^7^ CCU per chicken) following a previously described method [31]. Additionally, an NDV/IBV live nasal vaccine (Wuhan Keqian Biology Co., Ltd) (30 μL per bird) was administered to increase the incidence and severity of lesions. The experimental design is shown in Figure 2(a).

### Specific IgG and sIgA levels

Serum IgG, jejunal, and bronchoalveolar lavage fluid IgA responses to RS01790, BMP, GrpE, RS00900, and RS00275 were determined by ELISA. Fourteen days after the second immunization, three chickens were randomly selected from each group to collect intestinal and bronchoalveolar lavage fluid after euthanasia. The jejunal and lung tissues of the chickens were washed twice with a total of 2 mL of cold PBS, respectively. After centrifugation at 800 *× g* for 10 min, the supernatants of the jejunal and bronchoalveolar lavage fluid samples were collected. The ELISA procedure followed the previous description [36], wherein 96-well plates were coated with 100 μl of RS01790, BMP, GrpE, RS00900, and RS00275 proteins (1 μg/mL) and left overnight at 4°C. After blocking with 5% milk in PBST for 3 h at 37°C, 100 μl of diluted serum (1:100) and jejunal and bronchoalveolar lavage fluid (1:50) were added to each well and incubated at 37°C for 2 h. The HRP-conjugated goat anti-chicken IgG and goat anti-chicken IgA (Abcam, Cambridge, UK) secondary antibodies (1:5000) were then incubated for 1.5 h at 37°C. Plates were developed with TMB (Solarbio, Beijing, China) and quenched with 5% H_2_SO_4_. Absorbance was measured at 450 nm using an automated ELISA plate reader. A sample was deemed positive if the ratio of the positive value (P) to the negative value (N) was greater than 2.1 (P/N > 2.1).

### Specific lymphocyte proliferation response assay

Fourteen days post-final immunization, spleens were collected from three randomly selected birds in each group. After euthanasia but before collecting jejunal and bronchoalveolar lavage fluid, splenic lymphocytes were obtained using a Spleen Lymphocyte Separation Medium (Solarbio, Beijing, China). The splenic lymphocytes were then resuspended in RPMI 1640 supplemented with 10% FBS and 1% penicillin/streptomycin. Next, the cells were inoculated in 96-well plates (5 × 10 [5] cells/well). They were incubated with five antigens (10 μg/mL; specific antigen stimulation), concanavalin A (ConA, 10 μg/mL) (positive control), and culture medium (negative control), respectively, at 37°C and 5% CO2 for 48 h. Proliferation responses were measured using cck-8 (Solarbio, Beijing, China). For this, 10 µl of CCK-8 solution was added to each well for 4 h, and OD_450_ values were measured. The cell stimulation index (SI) was calculated according to the formula (OD _experiment_)/(OD _negative_) [37].

### Quantitative real-time PCR (qTR-PCR) for cytokines

To evaluate mRNA levels of IL-4, IL-17A, and IFN-γ expression after restimulation, splenic lymphocytes were inoculated in 24-well plates (5 × 10 [6] cells/well). The processing of cells is consistent with that described above. Total RNA extraction, cDNA transcription, and qRT-PCR were performed according to the previously described methods [31]. The primer sequences are shown in Table 2. The mRNA expression levels of IL-4, IL-17A, and IFN-γ were analyzed using the 2^‐ΔΔct^ method [38,39].

### Assessment of protective effectiveness

Protective efficacies were evaluated based on body weight gains, air sac lesions, tracheal mucosa thickness, and bacterial load. The body weight gain was presented as the difference in body weights between day 14 and day 0 post-challenge. The scoring system for air sac lesions used criteria previously described [12,40,41]. The average thickness of the upper, middle, and lower tracheal mucosa was measured at four points where horizontal and vertical lines intersected. Subsequently, these measurements were averaged. The quantification of *M. synoviae* load in laryngeal swabs was performed using qRT-PCR, as described previously [42]. To calculate the amount in each sample, CT values were converted to titers utilizing a standard curve made by known amounts of *M. synoviae* plasmid.

### Statistical analysis

Data were analyzed by one-way analysis of variance (ANOVA) using Microsoft Excel and Prism 8.0 (GraphPad). *p* < 0.05 was considered to be statistically significant. All data were expressed as the mean ± SD.

## Results

### Construction and characterization of the RASV strains

**Figure 1.**
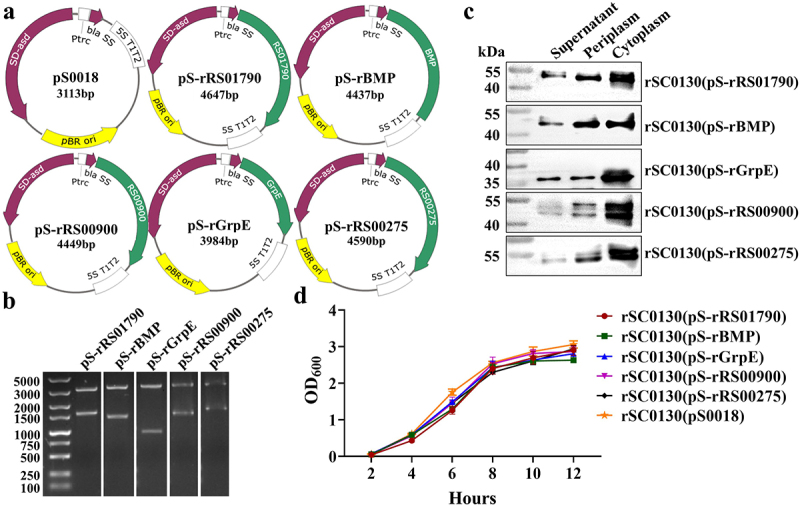
Plasmid maps and construction results, phenotypic characteristics of vaccine candidate strains. (a) Plasmid maps of pS0018, pS-rRS01790, pS-rBMP, pS-rGrpe, pS-rRS00900, and pS-rRS00275. (b) Identification of recombinant plasmid pS-rRS01790, pS-rBMP, pS-rGrpe, pS-rRS00900, and pS-rRS00275 by restriction enzyme digestion. *EcoR* I and *Sal* I digested pS-rRS01790, pS-rBMP, pS-rGrpe, pS-rRS00900, and pS-rRS00275. (c) The expression of rRS01790, rBMP, rGrpe, rRS00900, and rRS00275 in rSC0130 was analyzed by western blot. (d) Growth curves of the rSC0130(pS-rRS01790), rSC0130(pS-rBMP), rSC0130(pS-rGrpe), rSC0130(pS-rRS00900), rSC0130(pS-rRS00275), and rSc0016(ps0018) strains in the LB medium.

The RS01790, BMP, GrpE, RS00900, and RS00275 genes of *M. synoviae* were cloned into the pS0018 (Asd^+^ plasmid) to form pS-rRS01790, pS-rBMP, pS-rGrpE, pS-rRS00900, and pS-rRS00275 (Figure 1(a)). The positive plasmids were identified by double enzyme digestion with the *EcoR* I and *Sal* I restriction enzymes (Figure 1(b)). Plasmids pS0018, pS-rRS01790, pS-rBMP, pS-rGrpE, pS-rRS00900, and pS-rRS00275 were introduced into the rSC0130 strain via electroporation. The presence of proteins in the supernatant fraction may be associated with the immune protection effect. Cytoplasm, periplasm, and culture supernatant fractions of rSC0130 (pS-rRS01790), rSC0130(pS-rBMP), rSC0130(pS-rGrpE), rSC0130(pS-rRS00900), and rSC0130(pS-rRS00275) were prepared to determine the localization of rRS01790, rBMP, rGrpE, rRS00900, and rRS00275 in the five vaccine strains. The results indicated that rRS01790, rBMP, rGrpE, rRS00900, and rRS00275 were produced in the three fractions of rSC0130(pS-rRS01790), rSC0130(pS-rBMP), rSC0130(pS-rGrpE), rSC0130(pS-rRS00900), and rSC0130(pS-rRS00275), respectively (Figure 1(c)).

Growth curve results indicated that rSC0130 (pS0018) demonstrated comparable growth characteristics to rSC0130(pS-rRS01790), rSC0130(pS-rBMP), rSC0130(pS-rGrpE), rSC0130(pS-rRS00900), and rSC0130(pS-rRS00275) (Figure 1(d)). These findings suggest that the introduction of heterologous antigens did not impede the growth of rSC0130. To measure the stability of the plasmids, rSC0130 containing plasmids pS-rRS01790, pS-rBMP, pS-rGrpE, pS-rRS00900, and pS-rRS00275 were cultured for 50 generations. PCR and the endonuclease digestion results showed that pS-rRS01790, pS-rBMP, pS-rGrpE, pS-rRS00900, and pS-rRS00275 could be stably maintained in rSC0130 vaccine strains (Figure S1).

### Antibody responses in chicken after immunization with the RASV strains

**Figure 2.**
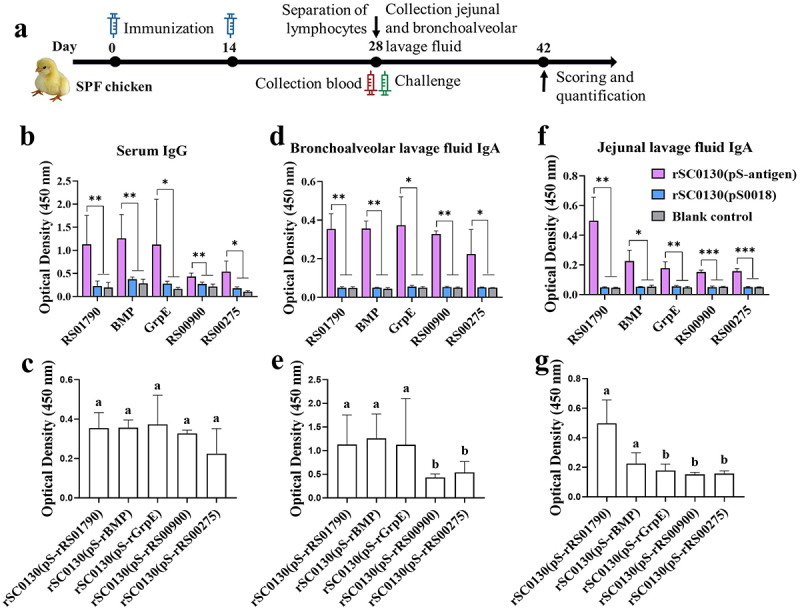
Scheme of immunization and challenge regimen and specific IgG and sIgA levels elicited by rSC0130(pS-rRS01790), rSC0130(pS-rBMP), rSC0130(pS-rGrpe), rSC0130(pS-rRS00900), rSC0130(pS-rRS00275), and rSc0016(ps0018) vaccines. (a) Schematic diagram of the immunization and challenge experiment in chicken. The chickens immunized intramuscularly two times with rSC0130(pS-rRS01790), rSC0130(pS-rBMP), rSC0130(pS-rGrpe), rSC0130(pS-rRS00900), rSC0130(pS-rRS00275), and rSc0016(ps0018) vaccines. They were then challenged with *M. synoviae* rSC0200 strain (9 × 10^7^ CCU) one week after the final immunization. The first immunization was administered when the chickens were one day old (0 weeks post immunization (0 wpi)), followed by booster immunizations at 15 days of age. At 29 days old, the chickens were challenged with *M. synoviae* and euthanasia was performed at 43 days old (6 wpi). The experiments were performed in triplicate. (b, c). The specific serum IgG levels determined by ELISA. (d, e). The specific IgA levels in bronchoalveolar lavage fluid determined by ELISA. (f, g). The specific IgA levels in jejunal lavage fluid determined by ELISA. Statistical analysis was performed using the Mann–Whitney U test (***p < 0.001, **p < 0.01, *p < 0.05, ns *p* ≥ 0.05). Error bar, mean ± S.D. Values within a column with different lowercase superscripts (a, b, c) are significantly different (*p* < 0.05) in figures c, e, and g.

To determine the responses induced by rSC0130 (pS-rRS01790), rSC0130(pS-rBMP), rSC0130(pS-rGrpE), rSC0130(pS-rRS00900), and rSC0130(pS-rRS00275), serum IgG, jejunal, and bronchoalveolar lavage fluid IgA were evaluated using ELISA. Vaccination with rSC0130(pS-rRS01790), rSC0130(pS-rBMP), rSC0130(pS-rGrpE), rSC0130(pS-rRS00900), and rSC0130(pS-rRS00275) resulted in higher specific serum IgG levels against RS01790, BMP, GrpE, RS00900, and RS00275 than those induced by rSC00(pS0018) and PBS group (Figure 2(b,c)). Inoculation with rSC0130 (pS-rRS01790), rSC0130(pS-rBMP), rSC0130(pS-rGrpE), rSC0130(pS-rRS00900), and rSC0130(pS-rRS00275) elicited greater specific bronchoalveolar lavage fluid IgA levels targeting RS01790, BMP, GrpE, RS00900, and RS00275, compared to levels induced by rSC0130(pS0018) and PBS group (Figure 2(d,e)). There was no significant difference between vaccine groups. Administration of rSC0130 (pS-rRS01790), rSC0130(pS-rBMP), rSC0130(pS-rGrpE), rSC0130(pS-rRS00900), and rSC0130(pS-rRS00275) resulted in higher specific jejunal lavage fluid IgA levels against RS01790, BMP, GrpE, RS00900, and RS00275, in contrast to levels induced by rSC01130(pS0018) and PBS group (Figure 2(f,g)). The levels of IgA in jejunal lavage fluid in the rSC0130(pS-rRS01790) and rSC0130(pS-rBMP) groups were significantly higher than those in the other three vaccine groups.

### Antigen-specific stimulation of significant cellular immune responses

**Figure 3.**
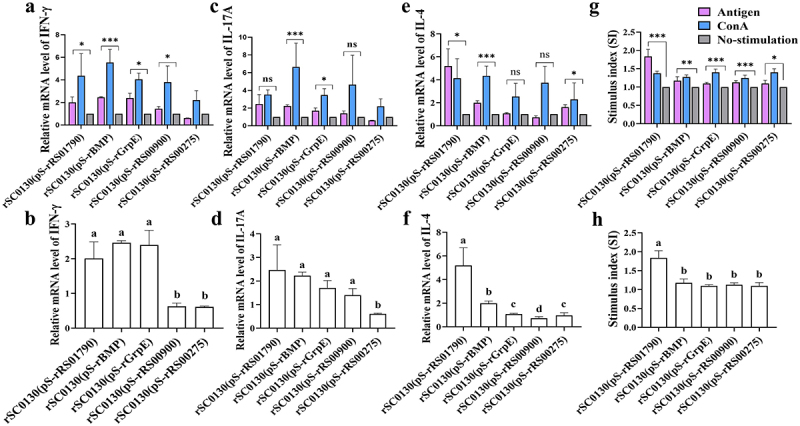
Cellular immunity response elicited by rSC0130(pS-rRS01790), rSC0130(pS-rBMP), rSC0130(pS-rGrpe), rSC0130(pS-rRS00900), rSC0130(pS-rRS00275), and rSc0016(ps0018) vaccines. (a-f) mRNA expression levels of IFN‐γ (a, b), IL‐17 a (c, d), and IL‐4 (e, f) in lymphocyte cells were quantified. (g, h) the proliferation response of chicken lymphocyte cells was detected by the CCK‐8 assay. Statistical analysis was performed using the Mann–Whitney U test (***p < 0.001, **p < 0.01, *p < 0.05, ns *p* ≥ 0.05). Error bar, mean ± S.D. Values within a column with different lowercase superscripts (a, b, c) are significantly different (*p* < 0.05) in figures b, d, f, and h.

The cellular-mediated immune response holds significance in clearing mycoplasma infections and might play a crucial role in vaccine-induced protective immunity [43]. Upon in vitro stimulation of cells with the five antigens (rRS01790, rBMP, rGrpE, rRS00900, and rRS00275), the results are shown in Figures 3(a-f). Stimulation with rRS01790 increased IFN-γ and IL-4 levels. Stimulation with rBMP increased IFN-γ, IL-17A, and IL-4 levels. Stimulation with rGrpE significantly increased IFN-γ and IL-17A levels. Stimulation with rRS00900 significantly increased IFN-γ levels only. However, rRS00275 stimulation led to an increase only in the mRNA level of IL-4. The CCK-8 assay was employed to assess specific lymphocyte proliferations in the spleen. Notably, all five vaccination groups displayed significantly higher lymphocyte proliferation levels after antigen treatment compared to the non-stimulated cells (Figure 3(g)). Among them, the cells in the rSC0130 (pS-rRS01790) immune group exhibited more pronounced cell proliferation after protein restimulation when compared to the other immune groups. However, the use of protein stimulation did not trigger cell proliferation in both the pS0018 group and the blank control group (Figure 3(h)).

### RASVs reduced clinical symptoms caused by M. synoviae infection and bacterial load

**Figure 4.**
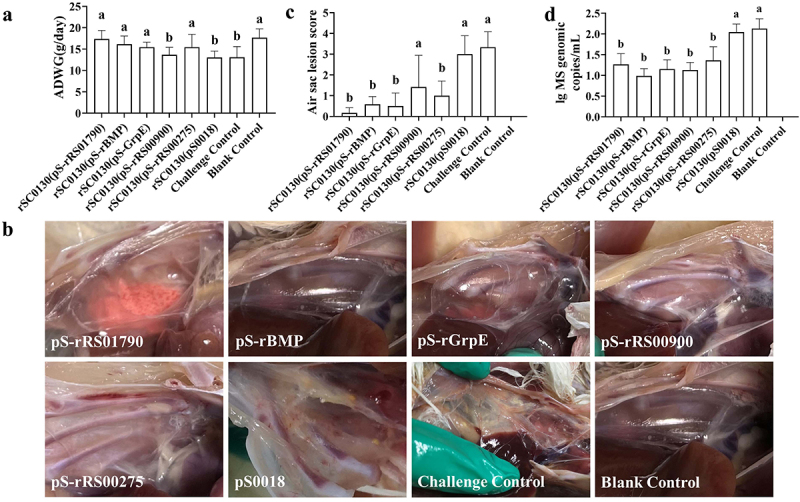
ADWG, gross air sac lesions, and *M. synoviae* loads in SPF chicken at 14 days post infection. (a) Results of average daily weight gain (ADWG) in chicken. (b) Air sac lesion scores in chicken at 14 days post infection among groups on a 5-point scale from 0 to 4 (0, No air sac lesion was observed; 1, slight cloudiness of the air sac membrane were found; 2, air sac membrane was slightly thick and usually presented small accumulations of cheesy exudates; 3, air sac membrane was obviously thick and meaty in consistency, with large accumulations of cheesy exudates in one air sac; 4, lesions were observed the same as 3, but two or more air sacs were found.). (b) Appearance of the air sac in chicken after 14 days of infection. (d) Microbial loads of *M. synoviae* in chicken pharynx swab measured by qRT-PCR method after being challenged with rSC0200 strain. Statistical analysis was performed using the Mann–Whitney U test. Values within a column with different lowercase superscripts (a, b) are significantly different (*p* < 0.05) in (a, b, and, d).

The protective efficacy of the vaccine against *M. synoviae* was evaluated based on parameters including body weight gains, bacterial load, air sac lesions, and tracheal mucosa thickness. Body weight gain is quantified using the metric of average daily weight gain (ADGW; grams/chicken/day). The ADGW for the (pS-rRS01790), (pS-rBMP), (pS-rGrpE), and (pS-rRS00275) vaccine groups exhibited a significant increase when compared to both the challenge group and the (pS0018) group, with the exception of the (pS-rRS00900) group (Figure 4(a)). Following immunization, the five vaccine groups partially alleviated the air sac inflammation and exudation caused by *M. synoviae* infection (Figure 4(b)). The air sac lesion scores of chickens immunized with the (pS-rRS01790), (pS-rBMP), (pS-rGrpE), and (pS-rRS00275) strains were significantly lower compared to the challenge groups and the (pS0018) group (Figure 4(c)). Although the average score of the air sac in the (pS-rRS00900) immune group was lower than that in the control group, the difference was not statistically significant. The average DNA copy number of positive samples in the (pS-rRS01790), (pS-rBMP), (pS-rGrpE), (pS-rRS00900), and (pS-rRS00275) groups was notably lower than that in the challenge group and the (pS0018) group. Moreover, the blank control group showed no detection of *M. synoviae* (Figure 4(d)).

### RASVs improved ameliorates microscopic tunica mucosa tracheae lesions in chicken

**Figure 5.**
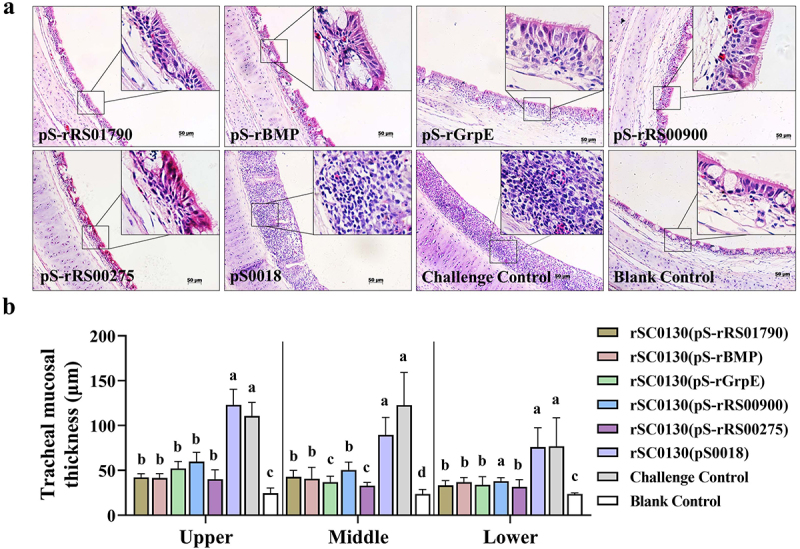
Pathological lesions of chicken tracheal mucosa tissue and mean tracheal mucosal thickness after *M. synoviae* challenge. (a) Tracheal mucosa tissue of the experimental chicken was sectioned and stained with H&E on day 14 after challenge with *M. synoviae*. Scale bar = 50 µm. (b) The mean tracheal mucosal thickness of the upper, middle and lower tracheas of the chicken on day 14 after challenge with *M. synoviae*. Statistical analysis was performed using the Mann–Whitney U test. Error bar, mean ± S.D. Values within a column with different lowercase superscripts (a, b, c) are significantly different (*p* < 0.05) in figure b.

The groups vaccinated with (pS-rRS01790), (pS-rBMP), (pS-rGrpE), and (pS-rRS00275), as well as the blank control group, displayed a pseudo-stratified ciliated columnar epithelium in the tracheal mucosa. This was accompanied by numerous simple intraepithelial mucous glands containing several goblet cells. In the tracheal mucosa from the (pS-rRS00900) vaccinated group, mild mucosal thickening was observed due to several lymphfollicular infiltrates. In contrast, chickens in the challenge group and those vaccinated with (pS0018) exhibited a lack of ciliated columnar epithelium and intraepithelial mucous glands in the tracheal mucosa. This was associated with lymphocytic infiltration, and the original columnar epithelium was substituted with a simple squamous epithelium, featuring both diffuse infiltration and focal aggregation of lymphocytes (Figure 5(a)). Concerning the mean thickness of the upper and middle tracheal mucosa, the five immunized groups displayed significantly reduced thickness compared to both the challenge control and the group vaccinated with (pS0018). However, their thickness was thicker than that observed in the blank control group. In the lower trachea, the tracheal mucosa of the (pS-rRS01790), (pS-rBMP), (pS-rGrpE), and (pS-rRS00275) immunized groups displayed significantly thinner mucosa compared to the challenge control and (pS0018) group. Nevertheless, no significant difference was noted in the (pS-rRS00900) vaccinated group when compared to both the challenge control and the group vaccinated with (pS0018) (Figure 5(b)).

## Discussion

Recent studies have shown an alarming rise in the prevalence of *M. synoviae*, leading to substantial economic losses in the poultry industry due to respiratory diseases, egg production decline, and increased culling [2,44–49]. Vaccination remains a crucial strategy to mitigate these impacts, and the development of safe, effective vaccines is an ongoing challenge. In this study, five recombinant attenuated Salmonella vaccines (RASVs) were constructed to express different *M. synoviae* antigens – RS01790, BMP, GrpE, RS00900, and RS00275. These candidate strains, namely rSC0130(pS-rRS01790), rSC0130(pS-rBMP), rSC0130(pS-rGrpE), rSC0130(pS-rRS00900), and rSC0130(pS-rRS00275), were evaluated for their growth characteristics, antigen expression, and immune-protective effects.

One important consideration in the development of live attenuated vaccines is the balance between antigen expression and bacterial fitness. Excessive metabolic burden due to exogenous antigen synthesis can potentially hinder the growth of the bacterial vector, reducing vaccine efficacy [28]. Our findings demonstrated that the growth curves of all five RASV strains were comparable to the control strain rSC0130(pS0018), indicating that antigen expression did not compromise bacterial fitness. This suggests that these candidate strains are suitable for use as live vaccines without negatively affecting their viability, an essential attribute for live attenuated vaccines where the bacteria must persist long enough to elicit robust immune responses [50].

In terms of immune responses, our results confirm that all five vaccine strains were capable of synthesizing and secreting the target antigens, which is critical for stimulating effective immunity. Antibody responses, particularly antigen-specific serum IgG and mucosal IgA, are indicative of successful antigen processing and presentation [51]. All five candidate vaccines induced these responses, which correlates with the ability of live vaccines to trigger both humoral and mucosal immunity. In comparison to inactivated vaccines, RASV generally induces strong mucosal immunity by colonizing lymphoid tissue [52]. Mucosal immunity is a critical first line of defense against respiratory pathogens such as *M. synoviae* [20].

Additionally, cellular immune responses were robust, with antigen-specific lymphocyte proliferation observed across all groups. This indicates that the vaccine strain rSC0130 successfully presented the foreign antigens. Notably, different vaccines stimulated distinct patterns of T helper (Th) cell responses, which can shape the quality and duration of immune protection. For instance, rSC0130(pS-rRS01790) induced both Th1 and Th2 responses, rSC0130(pS-rBMP) induced Th1, Th2, and Th17 responses, and rSC0130(pS-rGrpE) induced Th1 and Th17 responses. The induction of multiple Th pathways, particularly the Th17 response, is advantageous as it promotes mucosal immunity and pathogen clearance at the respiratory surface, where *M. synoviae* primarily infects [53]. This differential activation of immune pathways may partly explain the variation in protective efficacy among the vaccine candidates.

Regarding immune protection, the rSC0130(pS-rRS01790), rSC0130(pS-rBMP), and rSC0130(pS-rGrpE) strains showed superior protection by significantly reducing *M. synoviae* load in the pharynx and preventing tracheal mucosal damage. These vaccines also improved recovery from infection-related growth performance loss. In contrast, rSC0130(pS-rRS00900) and rSC0130(pS-rRS00275) demonstrated less pronounced protection, with the latter group showing slower recovery and no significant difference in air sac scores compared to the control. These findings suggest that while the five candidate vaccines show promise, variations in immune response and protection highlight the need for further optimization. One potential explanation for these variations could be differences in antigen presentation or immune response modulation by the different antigens. Further research is needed to elucidate the molecular mechanisms behind these differential responses, such as antigen stability, expression levels, or the intrinsic immunogenicity of each antigen. Investigating these factors in future studies could guide improvements in vaccine design to enhance protective efficacy.

In conclusion, rSC0130(pS-rRS01790), rSC0130(pS-rBMP), and rSC0130(pS-rGrpE) demonstrated strong protective effects in the SPF chicken model, effectively reducing *M. synoviae* infection and related pathology. These findings emphasize the potential of RASVs expressing *M. synoviae* antigens as viable vaccine candidates. The ability of these vaccines to elicit both humoral and cellular immune responses provides a solid foundation for further development, offering valuable insights into the design of future *M. synoviae* vaccines for poultry.

## Authors statement

All authors declare that the work presented in this manuscript is original and has not been published elsewhere. The authors also provided approval of the revised version to be submitted and take full responsibility for the content and integrity of the manuscript.

## Supplementary Material

FigureS1.jpg

Supplementary_Information - QVIR-2024-0132.R3.docx

## Data Availability

The data that support the findings of this study are openly available in Figshare at https://doi.org/10.6084/m9.figshare.25357006.

## Statement

In our manuscript, we have adhered to the ARRIVE guidelines.
