# Supplementary figures and images for: Construction and protective evaluation of a recombinant attenuated Salmonella vaccine delivering Mycoplasma synoviae antigens
Source: Virulence. 2025 Aug 8;16(1):2545554. doi: 10.1080/21505594.2025.2545554 (PMC12341054; doi:10.1080/21505594.2025.2545554)

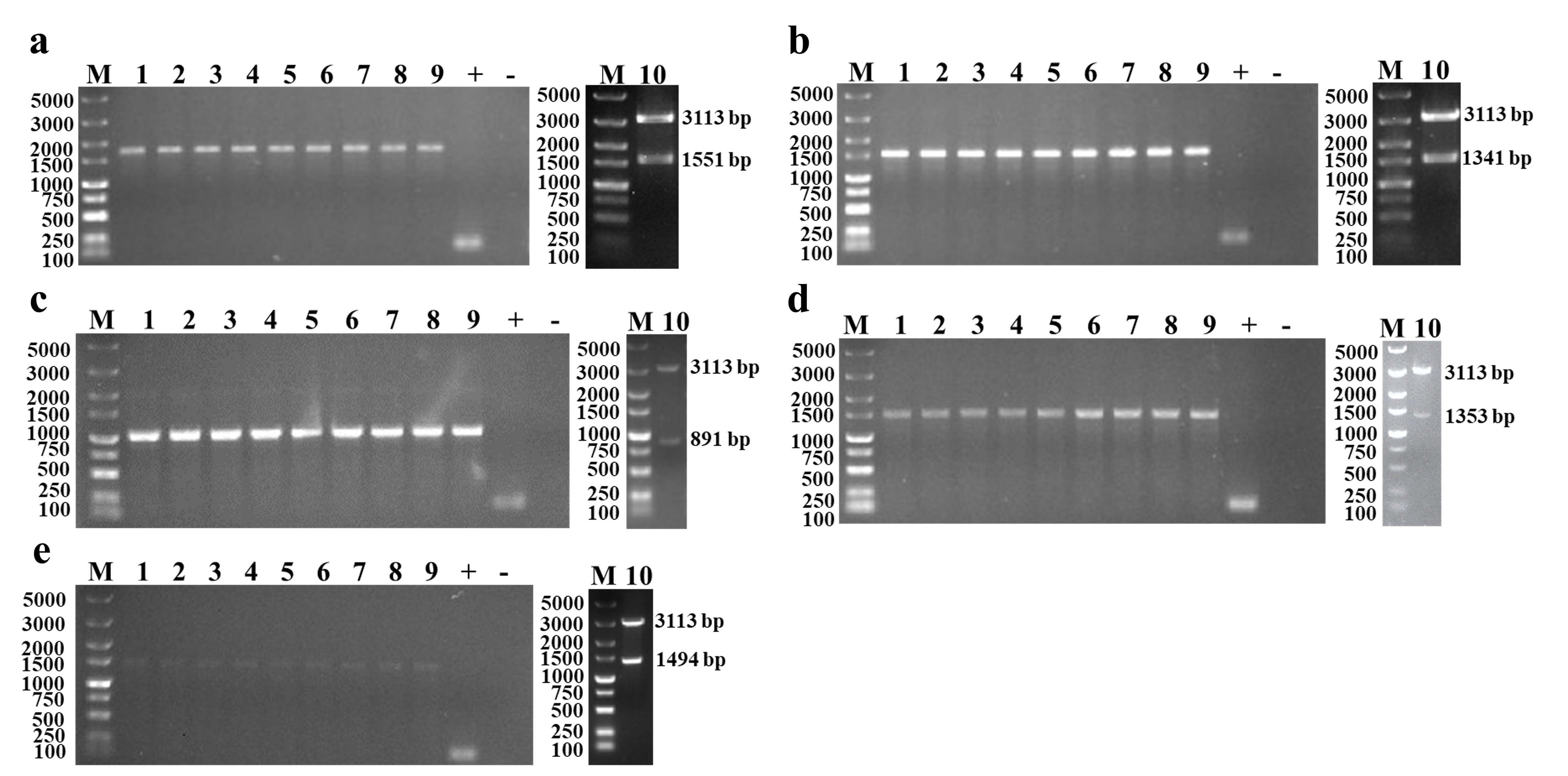

Supplement: FigureS1.jpg [file KVIR_A_2545554_SM3829.jpg]
