# Supplementary material for: Construction and protective evaluation of a recombinant attenuated Salmonella vaccine delivering Mycoplasma synoviae antigens
Source: Virulence. 2025 Aug 8;16(1):2545554. doi: 10.1080/21505594.2025.2545554 (PMC12341054; doi:10.1080/21505594.2025.2545554)
Supplement: Supplementary_Information - QVIR-2024-0132.R3.docx [file KVIR_A_2545554_SM3828.docx]

**SUPPLEMENTARY FIGURES**


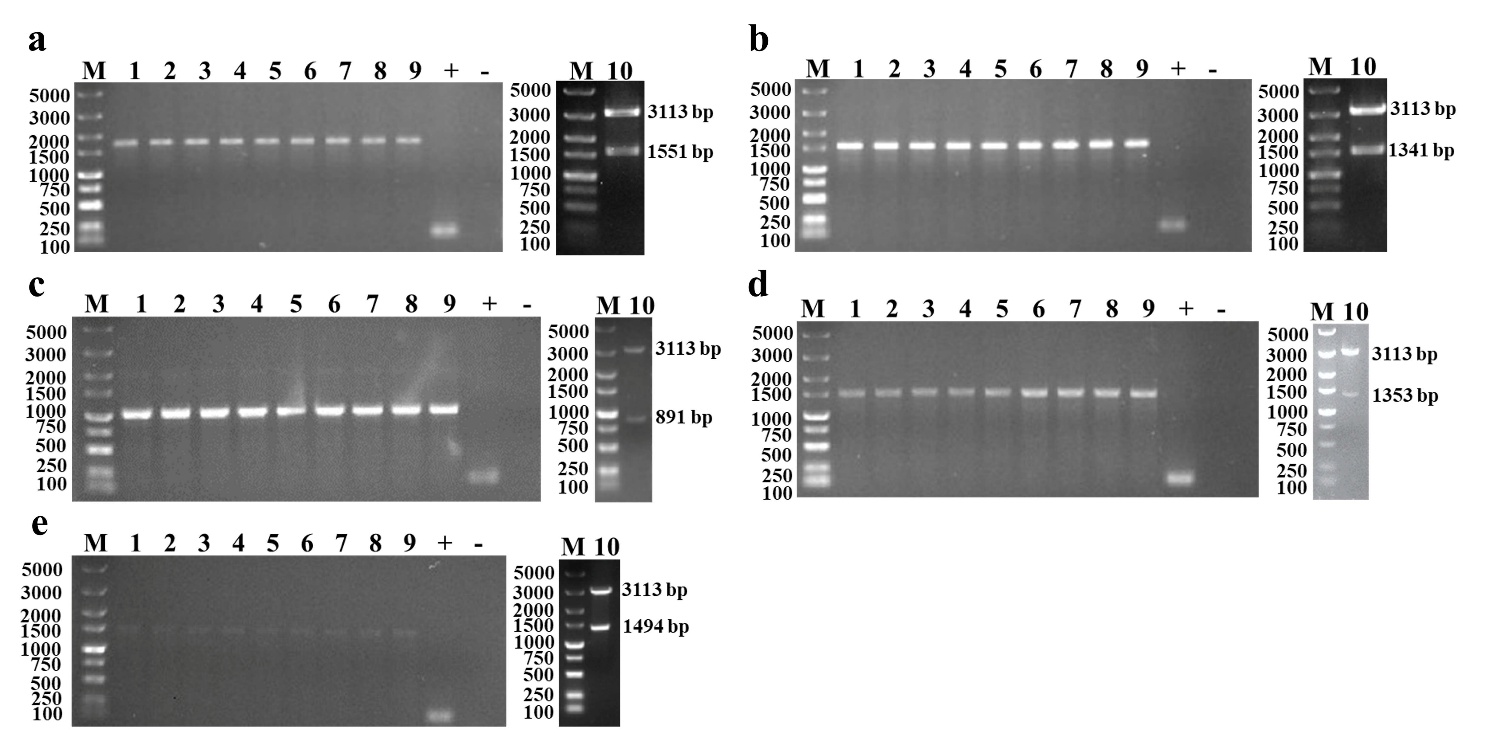


**Figure S1**. Plasmid stability of pS-Rrs01790 (a), pS-rBMP (b), pS-rGrpE (c), pS-rRS00900 (d), and pS-rRS00275 (e) during passage. M: DL5000 DNA marker; Lane 1-9: Colony PCR identification results; lane +: Positive control (pS0018 plasmid); lane -: Negative control (H2O); lane 10: Plasmid double enzyme digestion results.
